# Precipitation Stripping of V(V) as a Novel Approach for the Preparation of Two-Dimensional Transition Metal Vanadates

**DOI:** 10.3390/nano14010038

**Published:** 2023-12-22

**Authors:** María Guadalupe Sánchez-Loredo, Paul Chekhonin, Doreen Ebert, Ulrike Fischer, Xu Liu, Robert Möckel, Gladis Judith Labrada-Delgado, Stefano Passerini, Norman Kelly

**Affiliations:** 1 Helmholtz-Zentrum Dresden-Rossendorf (HZDR), Helmholtz-Institut Freiberg für Ressourcentechnologie (HIF), Chemnitzer Str. 40, 09599 Freiberg, Germany; d.ebert@hzdr.de (D.E.); u.fischer@hzdr.de (U.F.); r.moeckel@hzdr.de (R.M.); n.kelly@hzdr.de (N.K.); 2 Instituto de Metalurgia, Facultad de Ingeniería, Universidad Autónoma de San Luis Potosí, Sierra Leona 550, San Luis Potosí 78210, Mexico; 3 Helmholtz-Zentrum Dresden-Rossendorf (HZDR), Institut für Ressourcenökologie, Bautzner Landstraße 400, 01328 Dresden, Germany; p.chekhonin@hzdr.de; 4 Helmholtz Institute Ulm (HIU), Helmholtzstraße 11, 89081 Ulm, Germany; xu.liu@kit.edu (X.L.); stefano.passerini@kit.edu (S.P.); 5 Karlsruhe Institute of Technology (KIT), P.O. Box 3640, 76021 Karlsruhe, Germany; 6 Instituto Potosino de Investigación Científica y Tecnológica, Camino a la Presa San José 2055, San Luis Potosí 78216, Mexico; gladis.labrada@ipicyt.edu.mx; 7 Chemistry Department, Sapienza University of Rome, Piazzale Aldo Moro 5, I-00185 Rome, Italy

**Keywords:** two-dimensional materials, transition metal vanadates, solvent extraction, precipitation stripping, cathode materials, batteries

## Abstract

Cobalt, nickel, manganese and zinc vanadates were synthesized by a hydrometallurgical two-phase method. The extraction of vanadium (V) ions from alkaline solution using Aliquat^®^ 336 was followed by the production of metal vanadates through precipitation stripping. Precipitation stripping was carried out using solutions of the corresponding metal ions (Ni (II), Co (II), Mn (II) and Zn (II), 0.05 mol/L in 4 mol/L NaCl), and the addition time of the strip solution was varied (0, 1 and 2 h). The time-dependent experiments showed a notable influence on the composition, structure, morphology and crystallinity of the two-dimensional vanadate products. Inspired by these findings, we selected two metallic vanadate products and studied their properties as alternative cathode materials for nonaqueous sodium and lithium metal batteries.

## 1. Introduction

Transition metal vanadates have shown potential in application as sensors, photo- and electrocatalysts, and recently for primary and rechargeable lithium-ion batteries (LIBs), mainly because of their high theoretical capacity, simple preparation, and low cost [1,2,3,4]. Vanadium oxides have for a long time attracted attention as promising electrode materials for batteries because of the multiple valence states of vanadium and rich structural chemistry, redox-dependent properties, and high theoretical capacities [2,4,5,6]. But vanadium oxides tend to become amorphous upon cycling, and for this reason, a second metal cation can be introduced to stabilize the framework [2].

Currently, various transition metal vanadates are employed as promising electrode materials for LIBs due to their layered crystal structure, active electrochemical properties, and good reversibility, and plenty of research work has been done on their synthesis and electrochemical properties [2,7]. Incorporation of another metal ion between the layers of vanadium oxide expands the interlayer space, enabling the effective intercalation/deintercalation of Li ions [8].

Nevertheless, metal vanadates are characterized by poor electrical conductivity and large volume expansion/contraction upon cycling, leading to limited rate performance and poor capacity stability [3]. To address these issues, an effective strategy is to develop two-dimensional (2D) nanosheet-based materials, with access of Li^+^, short ion diffusion pathways, and abundant electrochemically active sites [3]. These kinds of material are normally synthesized by hydrothermal methods at high temperatures and pressures, making the synthesis expensive, energy consuming, and the control of the microstructure and composition difficult to achieve [9,10,11].

Vanadium can currently be found in by-products of metallurgical processes, and in spent catalysts used by the chemical industry. The development of efficient techniques for the recovery of the metal and its purification is therefore relevant. In this work, different metal vanadate nanostructures are synthesized by an uncomplicated synthetic route by extracting V(V) with an organic solvent, followed by precipitation stripping. Cobalt, nickel, manganese and zinc were selected as the precipitating metal owing to the wide range of applications of their corresponding vanadate compounds.

The modification of the conventional hydrometallurgical process known as solvent extraction consists of the addition of a crystallization operation directly from the organic phase from the typical solvent extraction, where low-solubility metal salts, such as oxides, oxalates, or sulfides, can be precipitated [12,13,14,15,16].

In this work, Aliquat^®^ 336 was expected to act as both an ion exchange reagent in the solvent extraction, and as a growth controlling agent during the subsequent precipitation stripping step. The extraction of V(V) with Aliquat^®^ 336 is a well-established process. Afterwards, during the stripping step, the chloride ions and the chlorocomplexes of the transition metal ions can migrate to the organic phase, displacing the vanadate ions from the extract. Then, the last react at the interphase with the metallic ions forming the sparingly soluble vanadate compounds. The obtained materials are separated, washed and chemically and structurally characterized. The properties as alternative cathode materials of two selected metallic vanadates for nonaqueous sodium and lithium metal batteries were also studied.

## 2. Materials and Methods

The chemical synthesis was performed extracting the V(V) ions (prepared as a 2 g/L solution using V_2_O_5_ (Alfa Aesar, Haverhill, MA, USA, 99.2%) and 0.1 mol/L NaOH (Merck Millipore Corp., Burlington, MA, USA, pellets, Reag. Ph. Eur.) with an organic phase composed of 10 vol% Aliquat^®^ 336 (AQ336, Alfa Aesar) and 5 vol% n-octanol (Alfa Aesar, 99%) diluted in kerosene (J.T. Baker 8387, light distillate with a low aromatic content). The extraction time was 1 h, and the phase volume ratio was 1:1. The V(V) concentration in the aqueous phase before and after extraction was measured by ICP-OES analysis using a PlasmaQuant PQ9000 spectrometer (Analytik Jena, Jena, Germany), with a cyclonic spray chamber (glass) and a seaspray TM nebulizer (glass), and the extraction yield was calculated from the mass balance. Argon was used as a plasma gas (12 L min^−1^) and also to nebulize the sample (nebulizer gasflow 0.5 L min^−1^). The power of the generator was 1300 W and the observation was in axial view. The system was calibrated with 5-point calibration (0.5–5 mg L^−1^) and Y was used as an internal standard. Standards for calibration were prepared using single element solution (1000 mg L^−1^) from Inorganic Ventures. Finally, the evaluation software was AspectPQ (Analytik Jena).

The crystallization stripping of V(V) from the loaded organic solutions was performed using strip solutions containing a M (II)-salt, where M (II) was Zn (II), Ni (II), Co (II) and Mn (II) (ZnCl_2_, VWR Chemicals, Radnor, PA, USA, Reag. Ph. Eur.; NiCl_2_∙6H_2_O, Alfa Aesar, 99.95%; CoSO_4_∙7H_2_O, Thermo Scientific (Waltham, MA, USA), 98%; MnCl_2_∙4H_2_O, VWR Chemicals, Reag. Ph. Eur.), in highly concentrated sodium chloride (VWR Chemicals, Ph. Eur.) solution (4 mol L^−1^ in water). The experiments were carried out by adding 100 mL aqueous strip solution to 100 mL V(V)-loaded organic solution at a constant rate: 0 h (immediate addition), 1 h total addition time, and 2 h total addition time. After addition of the strip solution, the stirring of both phases continued for 1 h. The precipitates were collected by centrifugation and washed with deionized water, water/ethanol mixtures (1:1), ethanol (Carl Roth, Karlsruhe, Germany, >96%) and acetone (VWR Chemicals, technical) 3 times each. Then, the final product was obtained after vacuum-drying for 72 h. 

The powders underwent comprehensive characterization through X-ray diffraction (XRD), X-ray fluorescence (XRF), field emission scanning electron microscopy (FESEM), and infrared spectroscopy (FTIR). X-ray diffractograms were acquired using a PANalytical Empyrean diffractometer (PANalytical, Almelo, The Netherlands), which is equipped with a PIXcel3D-Medipix area detector (combined with a Fe-filter) and a Co X-ray tube (Kα = 1.789010 Å). Measurements were performed at a voltage of 35 kV and current of 35 mA, a scan range of 5–80° 2θ with a step size of 0.0131° resulting in an overall measurement time of approximately 10 h. The irradiated area on the samples was maintained at a constant 10 × 12 mm^2^ by means of an automatic divergence slit. Depending on the available amounts, the samples were either prepared in Si-sample holders or via the backloading technique. Qualitative data analysis was performed using the Highscore software 3.0.4 (PANalytical) and the pdf4+ database. Quantifications were carried out utilizing the so-called Rietveld method through the BGMN/Profex software package v. 5.0 [17,18]. The Rietveld refinement method of the structure was used to obtain the structural parameters of the phases that constitute the samples.

SEM micrographs were obtained using secondary electron imaging in an EVO 50 SEM (Zeiss, Jena, Germany), located at HZDR, Dresden-Rossendorf, equipped with a tungsten filament and operated at 15 kV acceleration voltage. The samples were prepared by suspending small amounts of the powders in ethyl alcohol, and the suspensions were evaporated on aluminum stubs acting as SEM sample holders. Energy dispersive X-ray spectroscopy (EDX) measurements were performed as point measurements using a Bruker EDX system.

Also, a small amount of each sample was dispersed in iso-propanol (Merck Millipore Corp, Supelco, Reag. Ph. Eur.) and subjected to sonication for 10 min. After that an aliquot was poured onto a copper grid, vacuum dried, and then, images using both backscattering and secondary electron detectors were obtained using Field Emission Scanning Electron Microscopy (FESEM) with a FIB Dualbeam FEI Helios 600 Nanolab Scanning Electron Microscope (Hillsboro, FL, USA), located at IPICyT (San Luis Potosí, Mexico).

Infrared spectra were obtained using a portable Cary 630 FTIR spectrometer (Agilent, Santa Clara, CA, USA) with an ATR unit and diamond as the optical material. Measurements were carried out at a temperature of 20 ± 2 °C with 32 scans per sample, 32 background scans with air as the background, a wavenumber range from 650 to 4000 cm^−1^ and a resolution of 2 cm^−1^. The spectra were presented in absorbance and the data were extracted from MicroLab Software (https://www.arturia.com, Agilent Technologies).

Electrochemical tests: The electrodes were fabricated by doctor-blade casting of slurries with 70 wt.% vanadate, 20 wt.% carbon black (Super C65, IMERYS), and 10 wt.% polyvinylidene fluoride binder (PVdF, Solef6020) on Al foil. After evaporating the NMP solvent at 80 °C in a Binder climatic chamber, the electrodes with a diameter of 12 mm were punched and further dried at 110 °C under vacuum (10^−3^ mbar) for 12 h. After being weighed in a dry room with a dew point < −60 °C and further dried at 110 °C under vacuum (10^−3^ mbar) for 4 h, the electrodes were transferred to an Ar-filled glove box with H_2_O and O_2_ levels of <0.1 ppm. The average mass loading of the active material was around 1.5 mg cm^−2^. Three-electrode T cells were assembled to evaluate the electrochemical performance of the electrodes as cathodes for lithium and sodium metal batteries. Glass fiber disks (Whatman GF/D, Maidstone, UK) were used as separators, and 150 μL electrolytes were injected for each cell. For the lithium metal batteries, lithium metal foils (99.9%, Honjo Metal, Osaka, Japan) were employed as the counter and reference electrodes, and 1 M LiPF_6_ in EC/DMC (1:1, *v*/*v*) provided by BASF was employed as the electrolyte. For the sodium metal batteries, sodium metal foils (Acros Organics, Geel, Belgium, 99%) were used as the counter and reference electrodes, and 1 M NaPF_6_ (FluoroChem, Glossop, UK, battery grade) in diethylene glycol dimethyl ether (diglyme, Sigma-Aldrich, St. Louis, MO, USA, anhydrous, 99.5%) was used as the electrolyte. The cells were tested at 20 °C with a MACCOR series 4000 battery cycler.

## 3. Results

**Figure 1:**
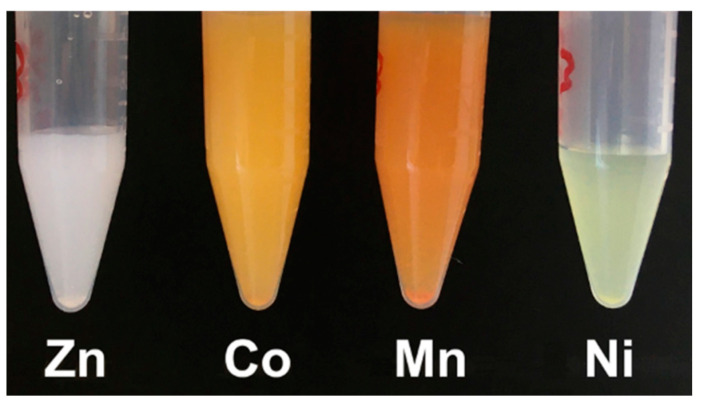
Metal vanadates suspensions in water.

A vanadium (V) extraction yield of 76.6% was obtained with the organic system consisting of Aliquat^®^ 336 20% (*v*/*v*)/1-octanol 10% (*v*/*v*)/kerosene and an aqueous phase containing 2 g L^−1^ V(V) in 0.1 N NaOH, corresponding to an organic phase loaded with 1.52 g L^−1^ V(V) [19]. After the extraction step, both phases were separated by centrifugation and the stripping solution (where the chloride ions act as the strip species and M (II) as the precipitation agent) was added to an aliquot of the organic phase under stirring, which produced finely dispersed powders, located in the aqueous phase and at the interphase. Centrifugation of the mixture allowed the separation of the obtained oily powders and both liquid phases. After washing, the powders were vacuum dried for structural and optical characterization. Figure 1 shows aqueous dispersions of the purified products.

**Figure 2:**
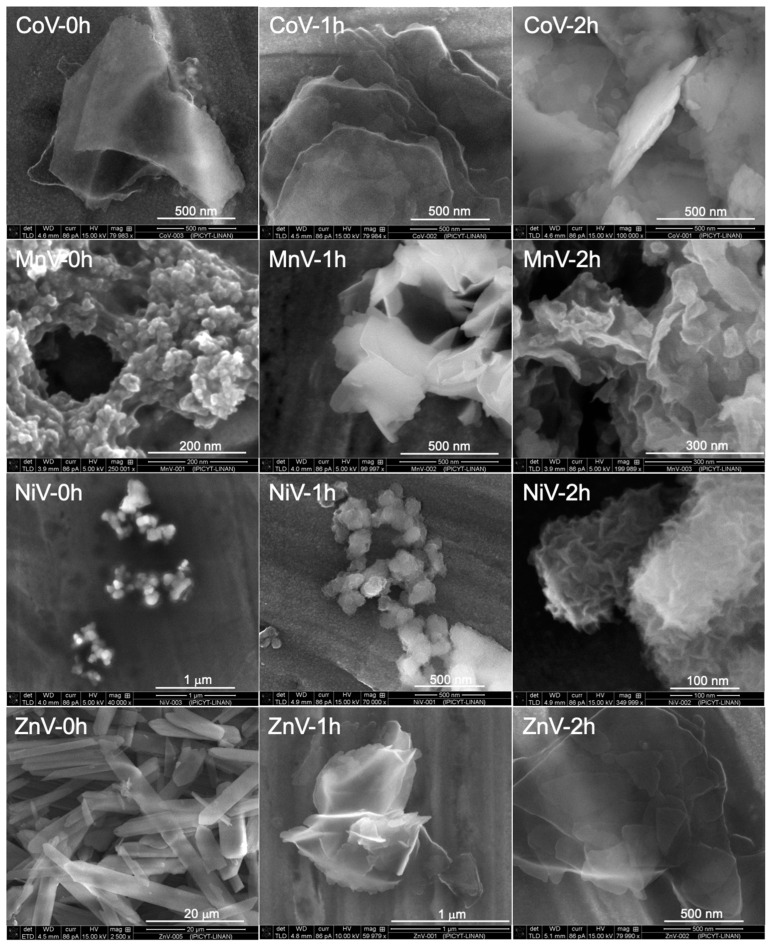
FESEM images of cobalt, manganese, nickel, and zinc vanadates obtained by precipitation stripping of V(V)-loaded organic solutions with M (II) solutions (0.05 M in 4 M NaCl). 0 h, 1 h, and 2 h corresponding to the time needed for addition of the aqueous to the organic solution.

High-resolution micrographs of the metallic vanadates are shown in Figure 2. When using Co (II) solutions as stripping and precipitation media, nanoplates of different morphologies and sizes were obtained. The sample obtained by rapid addition shows particles of different morphologies (ribbons, hexagon-like and irregular plates) with sizes varying from 1 to 5 microns, and thicknesses between 6 and 10 nm. SEM images show that the plates obtained with addition time of 1 h consist of rather rough quasi-spherical particles fused together. Longer addition times (2 h) produced particles of smaller size (200–300 nm) and thicknesses of around 20 nm.

For nickel, the rapid addition led to the formation of quasi-spherical structures, with a size range 50 to 150 nm, and these aggregates were formed by spherical particles of less than 10 nm. Longer addition times produced also similar structures, but a closer look shows that these aggregates were formed by nanoflakes of 2 nm thickness.

For stripping using manganese solutions, rapid addition gave place to porous nanostructures formed by the fusion of particles of sizes smaller than 20 nm. The pores formed varied from 40 to 150 nm. Longer addition times promoted the formation of flakes of 400 nm and thickness of 10 nm. The nanostructures obtained after 2 h addition time were composed of both flakes and quasi-spherical particles. The FESEM images in Figure 2 reveal closely connected nanoparticles that tended to form nanoscale voids and a well-defined sponge-like network. These nanostructures of fine grains, interweaved forming a three-dimensional network with abundant interspaces, could promote the diffusion of electrolyte and reactant ions to improve the electrochemical performance in batteries. Interesting was the morphology of the sample obtained at 1 h addition time; the nanosheets surface was very smooth, which could be ascribed to Ostwald ripening [3].

Figure 2 also shows images of samples obtained for 0 h, 1 h and 2 h addition time during stripping using zinc solutions. Increasing the addition time is an important parameter, particularly for stripping with Zinc (II) chloride solutions, affecting the chemical composition, morphology, and aggregation degree of the obtained vanadates. The preparation of the desired compounds was achieved only at longer addition times, otherwise, precipitation takes place after a few hours, and the sword-like microparticles (ZnV-0h), consisting of sodium vanadate, were obtained, as shown by EDS. In Figure S1 all EDS spectra of the particles are presented with the presence of vanadium and the corresponding metal in the precipitates, but in different proportions (copper arises from the SEM sample holder).

**Figure 3:**
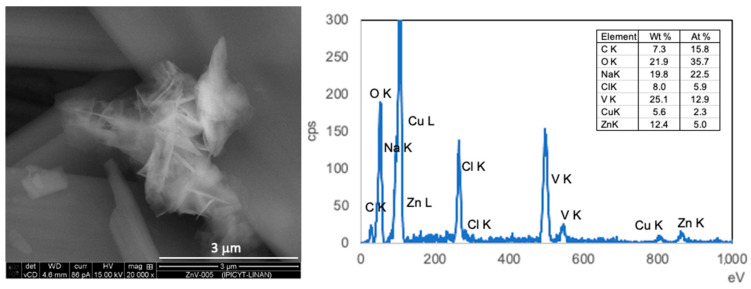
FESEM image and EDS spectrum of material obtained by precipitation stripping of V(V)-loaded organic solutions with Zn (II) solution (0.05 M in 4 M NaCl). 0 h corresponds to the time applied for addition of the aqueous to the organic solution.

Nevertheless, some isolated particles of zinc vanadate (Figure 3) with a morphology like the materials obtained after addition lasting 1 h and 2 h, are observed. The as-obtained zinc vanadates exhibit a uniform flower-like structure with an average size of 500 nm and petals with a width of few nm. The higher magnification images show that the flowers are assembled by nanosheets with smooth surfaces.

**Figure 4:**
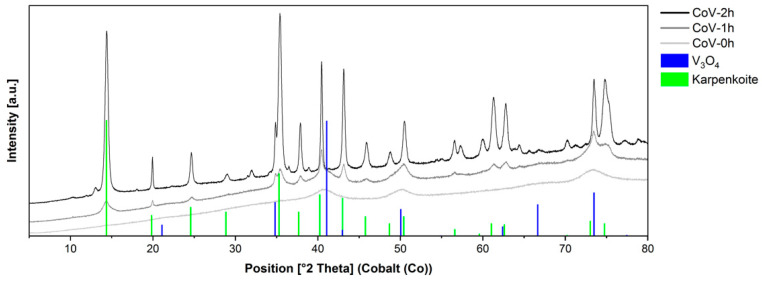
Diffractograms of cobalt vanadate samples at different addition times (0 h, 1 h, 2 h, uncorrected XRD diffractograms).

Based on the structural characterization of the materials with X-ray diffraction (XRD), Figure 4 highlights the XRD patterns of the materials obtained from the precipitation stripping of V(V) using a Co (II)-solution, at the three different addition times. Samples at longer additions times show the presence of karpenkoite, Co_3_(V_2_O_7_)(OH)_2_·2H_2_O, a cobalt analogue of martyite, trigonal, space group P-3m1. Immediate addition produced an amorphous material, and some vanadium oxide. On the contrary, slower addition led to purer materials and higher crystallinity. EDS element composition (Figure S1) indicated that the Co (II)/V atomic ratios were in good agreement (1.4 for CoV-1h and 1.5 for CoV-2h) with the empirical Co (II)/V atomic ratio of 1.5:1 for the chemical composition of Co_3_V_2_O_7_(OH)_2_.2H_2_O, whereas, the Co (II)/V atomic ratio for CoV-0h is 1:1.

The diffraction planes of the sample obtained over the reaction time of 1 h could be well indexed to cubic V_3_O_4_ (Spacegroup No = 227, HermannMauguin = F4_1/d-32/m), as well as to the trigonal crystal form of karpenkoite, Co_3_(V_2_O_7_)(OH)_2_·2H_2_O, a cobalt analogue of martyite, Zn_3_(V_2_O_7_)(OH)_2_(H_2_O)_2_ [20], space group 164 (P-3m1). The mineral karpenkoite can be seen as orange lamellar crystals up to 0.05 mm, coarsely hexagonal or irregular in shape, and the crystals, usually curved, grow in rose-like clusters or globular aggregates. This indicates the good incorporation of vanadate ions into the cobalt lattice. Rietveld refinement using the software Profex v5.1 estimated a karpenkoite content of 32.6%

**Figure 5:**
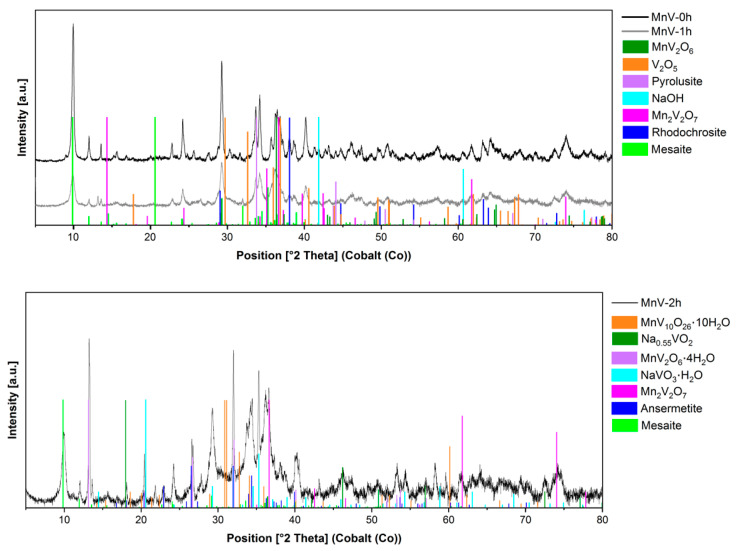
Diffractograms of manganese vanadate samples at different addition times (0 h, 1 h, 2 h).

When using Mn(II) salts as stripping agents, complex mixtures of materials containing manganese vanadates and oxides, and other compounds, were produced (Figure 5). MnV_2_O_6_, the hydrated ansermetite (Mn(V_2_O_6_)·4H_2_O), Mn_2_V_2_O_7_, Mn(V_10_O_26_)·10H_2_O, among other vanadates, were obtained. The presence of pyrolusite gives a hint that the Mn (II)-ions could act as reducing agents in the presence of V(V). The complexity of the mixtures obtained by using this technique makes this synthetic route inadequate for the preparation single-phase manganese vanadates.

XRD analysis of the nickel vanadate materials was also performed, and the XRD pattern corresponding to the sample NiV-1h is presented in Figure S2. As can be seen from the diffractogram, the Ni compound had a completely amorphous phase, and therefore, no Rietveld refinement could be performed for this material. In regard to the use of nickel solutions as stripping reagents, for the sample obtained for rapid addition, the V/Ni ratio obtained from the EDS quantification was 1.9, for 1 h addition, which corresponds to 1.3, whereas for 2 h, the V/Ni molar ratio was 1.5.

**Figure 6:**
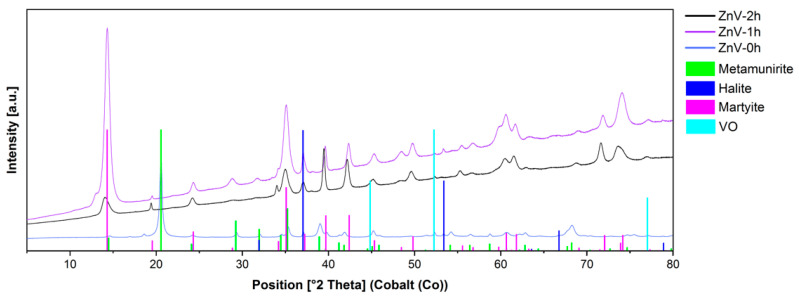
Diffractograms of samples obtained from stripping with Zinc (II) solutions at different addition times (0 h, 1 h, 2 h, uncorrected XRD diffractograms).

Figure 6 shows the typical XRD pattern of the as-prepared zinc vanadate materials. For 1 h and 2 h, the diffraction peaks can be readily indexed to the pure phase of Zn_3_(V_2_O_7_)(OH)_2_·2H_2_O, martyite, trigonal, space group P-3m1. On the contrary, for short addition time, the formation of NaVO_3_ (metamunirite, orthorhombic) was confirmed.

Rietveld refinement using Profex showed the presence of Zn_3_(VO_4_)_2_·3H_2_O. The diffraction peaks are indexed in a hexagonal unit cell, similar to the reported unit cell by Hoyos et al. [5] (ICSD ID 70264, Code 94034, space group P-6 with a = 6.07877 (8), c = 7.1827 (2) Å). The reported structure consists of vanadium tetrahedra bonded to distorted octahedral zinc atoms forming dimensional columnar passageways.

No peaks from other phases were detected for the samples obtained at longer addition times, giving a hint about the purity of the product. The byproducts observed with the sample at 1 h are salts that could be removed by washing, and the content was less than 3%, according to Rietveld refinement.

**Figure 7:**
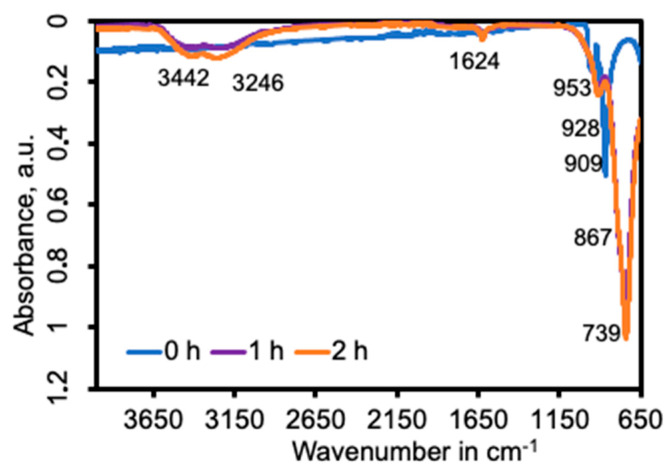
ATR spectra of zinc vanadate powders prepared by precipitation stripping from Aliquat 336 organic solutions.

To find out if Aliquat^®^ 336 (Figure S3) was acting as a stabilizer and covering the materials, ATR measurements were carried out at the solid samples (for zinc vanadates, Figure 7, for the other samples, Figure S4), and the spectra were compared with the spectrum of the quaternary ammonium salt, where the strong bands centered at 2852 cm^−1^ and 2921 cm^−1^ (corresponding to symmetric and asymmetric sp^3^ C-H stretching), 1465 cm^−1^ ((CH_3_)-N^+^), 1377 cm^−1^ (CH_3_ bending), 722 cm^−1^ (in-plane rocking mode of the methylene chain) [21,22,23] are all absent in the IR spectra. This could indicate that the extractant was not permanently attached to the surface of the particles, but its absence could also be a result of the washing with ethanol step.

In general, for all materials obtained by means of stripping using metal (II) solutions, ATR measurements carried out on the solid samples showed no bands corresponding to the spectrum of Aliquat^®^ 336. For cobalt, signals [11] reported for the characteristic absorption peaks for metal vanadates could be found at 885 and 1012 cm^−1^ (V-O-V and V-O vibrations, respectively). The bands at 764 cm^−1^ (2 h) and 774 cm^−1^ (1 h) can be assigned to the asymmetric vibrations of V-O-Co and V-O-V. Strong absorption peaks at 3518 and 1609 cm^−1^ are ascribed to the symmetric stretching and bending vibrations of H-O-H in H_2_O molecules belonging to the karpenkoite structure, respectively [21]. The broad peak at 3025 cm^−1^ can be ascribed to the OH group in the framework of karpenkoite.

The FTIR spectra of the manganese vanadate samples show an absorption band at 3486 cm^−1^ due to –OH stretching frequency [21]. The absorption band at 905 cm^−1^ corresponds to the symmetric stretching of terminal VO_3_ units, and the antisymmetric stretching of bridging V-O-V units appears at 772 cm^−1^ (0 and 1 h) and 782 cm^−1^ (2 h).

For nickel (II)-solutions as stripping agents, the IR spectra look similar to the one reported by Bülbü et al. [24] for Ni_3_(OH)_2_V_2_O_7_._5_·6H_2_O, but the positions of the XRD peaks for the samples are different from the compound reported. Nevertheless, the IR spectra indicate the presence of water molecules in the amorphous structures obtained. Again, the signals [11] reported for the characteristic absorption peaks for metal vanadates could be found at 885 and 1012 cm^−1^ (V-O-V and V-O vibrations, respectively). The band at 748 cm^−1^ (0 and 1 h) can be assigned to the asymmetric vibrations of V-O-Ni and V-O-V. The strong absorption peaks at 3324–3335 cm^−1^ and 1617 cm^−1^ correspond to the vibrations of H_2_O molecules [25].

ATR spectra of the zinc vanadate samples are depicted in the wavelength region of 650–4000 cm^−1^ in Figure 7. While the spectral shapes of the samples at 1 h and 2 h are similar, the intensities of the peaks are slightly different, on the contrary, the spectrum of 0 h shows different peak locations due to the different metals accompanying the vanadate moiety (Na or Zn). As for the other vanadates, the absorption peak at 739 cm^−1^ corresponds to V–O–Zn and V-O-V asymmetric vibrations [11]. The absorption bands at 3442 cm^−1^ and 1624 cm^−1^ are assigned to the vibration of water molecules in Zn_3_(VO_4_)_2_·3H_2_O. These signals are absent in sample 0 h, composed of sodium vanadate. The absorption band at 1621 cm^−1^ is assigned to the vibration of water molecules.

According to the literature, micro- and nanostructured vanadate products are almost exclusively synthesized by hydrothermal routes, normally with the assistance of organic templates or inorganic additives. This increases production costs and makes the scale-up difficult [26]. In this work, several vanadates of different morphologies and sizes were obtained by a simple hydrometallurgical process, but for manganese, the obtention of mixtures of vanadates and oxides could not be avoided. Optimization of the different operation parameter could allow for obtaining single phase products, and tailoring of the morphology and size of the obtained materials. In all cases, the large surface area of the synthesized vanadates provided a potential capacity for guest ion intercalation, fast ion diffusion, and charge transfer along open spaces and channels in the nanostructures.

Based on FESEM observations, the formation mechanism of hierarchical nanostructures could be as follows (but more experiments are needed in order to confirm this assumption): first, the vanadate ions are released from the organic phase and react with the metal ions forming tiny nuclei due to the low solubility product of the metal vanadates. The driving force for this release of vanadate ions is given by the stripping conditions applied, in this work, by the controlled flow of solution containing the metal and an important amount of chloride ions (probably the stripping agents). Due to the layered crystal structure and anisotropic growth habit of the metal vanadates, the nuclei develop in the aqueous solution system into nanoparticles and after that into nanosheets, where these then self-assemble into nanostructures to reduce the surface tension [26]. A secondary growth may take place if new crystals nucleate and grow on the surface of the nanosheets which then act as secondary nucleation sites. Thus, the nanostructures grow on the surfaces of the primary nanosheets. The hierarchical structures thus can be formed through a nucleation–anisotropic growth–self-assembly–secondary growth process [5].

Finally, two metallic vanadate products were selected in order to study their properties as alternative cathode materials for sodium and lithium metal batteries. The materials selected were the zinc vanadate obtained for the experiment of 1 h addition time, and the other a copper vanadate (volborthite), prepared according to a previous publication also for 1 h addition time [19].

**Figure 8:**
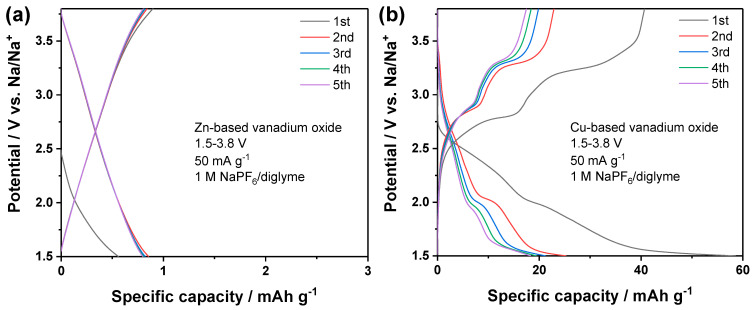
Dis-/charge profiles of (**a**) Zn-based vanadate and (**b**) Cu-based vanadate cathodes in sodium metal batteries employing 1 M NaPF_6_ in diglyme.4.

In a previous work, it was demonstrated that the ether-based electrolyte, 1 M NaPF_6_ in diglyme, has good interfacial compatibility towards both sodium metal anodes and vanadium oxide cathodes [27]. Therefore, the two vanadates were firstly evaluated as cathode materials for sodium metal batteries. The cells were dis-/charged at 50 mA g^−1^ within the potential window of 1.5–3.8 V vs. Na/Na^+^. The potential profiles are displayed in Figure 8. The zinc vanadate did not show any potential plateau and delivered nearly ignorable capacity, which suggests that this material is not able to store Na^+^ in this electrolyte. The Cu-based electrode showed two potential plateaus in the dis-/charge profiles, respectively, which indicates its electrochemical activity. In the initial cycle, the reversible capacity was around 40 mAh g^−1^, while the capacity delivered in the following cycles was around 20 mAh g^−1^. Obviously, Cu-based vanadate shows better electrochemical activity than Zn-based vanadate, which can be explained by the redox activity of Cu^2+^ and the catalytic effect of copper [28,29,30]. Despite the electrochemical activity, the specific capacity delivered by the Cu-based vanadate was too low for more practical application.

**Figure 9:**
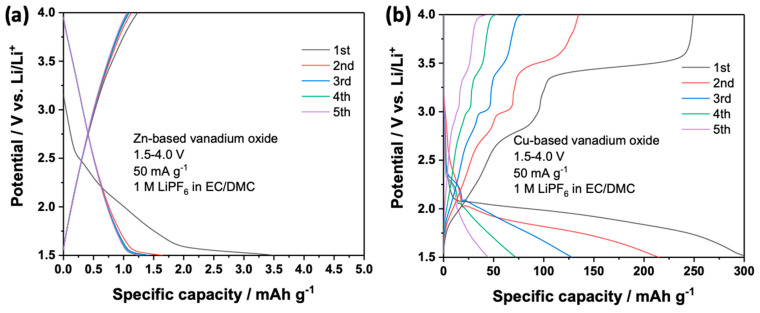
Dis-/charge profiles of (**a**) Zn-based vanadate and (**b**) Cu-based vanadate in lithium metal batteries employing 1 M LiPF_6_ in EC/DMC (1:1, *v*/*v*).

Employing Li^+^, with a smaller radius than Na^+^, as the charge carrier, could lead to promoted electrochemical activity. Therefore, these two materials were subjected to the lithium metal batteries tests. With conventional carbonate electrolytes, i.e., 1 M LiPF_6_ in EC/DMC (1:1, *v*/*v*), the cells were assembled and tested at 50 mA g^−1^ in the potential window of 1.5–4.0 V vs. Li/Li^+^. The voltage profiles upon dis-/charge are shown in Figure 9. The Zn-based vanadate still showed nearly zero capacity, which is the same as the previous run in sodium metal batteries. Therefore, Zn-based vanadate is electrochemically inactive in the tested potential window. The Cu-based vanadate showed again potential plateaus, which verifies its electrochemical activity. Due to the smaller radius of Li^+^ than Na^+^, this material exhibited much higher reversible capacity in the initial cycle, around 250 mAh g^−1^. Since these two materials exhibit a rather similar crystal structure, the higher electrochemical activity of Cu-based vanadate could be related to the replacement of Zn^2+^ by Cu^2+^. Unfortunately, a fast capacity fading was observed in the subsequent cycles. The specific capacity decreased to around 50 mAh g^−1^ at the 5th cycle. This could be related to the instability of the crystal structure upon Li^+^ de-/insertion and/or insufficiently protective cathode electrolyte interphase generated on Cu-based vanadate in the conventional carbonate-based electrolytes [31]. Further work is indeed required to promote the cyclability from the aspects of structural and morphological optimization of materials, as well as electrolyte engineering. Nonetheless, this result has demonstrated the possibility to gain an electrochemically active vanadate as electrode materials for potential battery application.

## 4. Conclusions

The obtained vanadate particles varied greatly in morphology, structure, crystallinity and size, ranging from ultrathin 2D nanosheets (cobalt vanadates, Mn vanadate, 1 h) to nanostructures assembled from quasi-spherical particles (manganese and nickel vanadates, rapid addition). Flower-like particles formed at longer addition times (Ni and Mn vanadates). However, pure zinc vanadate cannot be obtained in reactions of a short duration. The formation of nanoflakes could be attributed to the interior layered crystal structure. However, a key issue is phase purity. Regardless of the synthesis methodology applied, some of the products were not phase-pure.

These results revealed the advantages of combining the selectivity of the solvent extraction process with the possibility of direct fabrication of high-value products by precipitation stripping, where the production of other types of nanoparticles and nanostructures is feasible. More experiments are needed in order to prepare materials that are useful for electrodes in batteries.

## Data Availability

Data are contained within the article and supplementary materials.

## Supplementary Materials

The following supporting information can be downloaded at: https://www.mdpi.com/article/10.3390/nano14010038/s1, Figure S1: EDS spectra of the metal vanadates obtained at different addition times; Figure S2: Diffractogram of sample obtained from stripping with a Nickel (II) solution at an addition time of (0 h, 1 h, 2 h); Figure S3. Structure of Aliquat^®^ 336; Figure S4. ATR spectra of cobalt, manganese and nickel vanadate powders prepared by precipitation stripping from Aliquat 336 organic solutions.

## References

[1] Mahmoud S.A., Bendary S.H., Salem A.A., Fouad O.A. (2019). Facile synthesis of high yield two dimensional zinc vanadate nanoflakes. SN Appl. Sci..

[2] Luo L., Fei Y., Chen K., Li D., Wang X., Wang Q., Wei Q., Qiao H. (2015). Facile synthesis of one-dimensional zinc vanadate nanofibers for high lithium storage anode material. J. Alloys Compd..

[3] Yan H., Luo Y., Xu X., He L., Tan J., Li Z., Hong X., He P., Mai L. (2017). Facile and scalable synthesis of Zn_3_V_2_O_7_(OH)_2_·2H_2_O microflowers as a high-performance anode for Lithium-Ion Batteries. ACS Appl. Mater. Interfaces.

[4] Hao M., Xiao M., Qian L., Miao Y. (2018). Synthesis of cobalt vanadium nanomaterials for efficient electrocatalysis of oxygen evolution. Front. Chem. Sci. Eng..

[5] Hoyos D.A., Echavarría A., Saldarriaga C. (2001). Synthesis and structure of a porous zinc vanadate, Zn_3_(VO_4_)_2_·3H_2_O. J. Mater. Sci..

[6] Cao H., Zheng Z., Meng J., Xiao X., Norby P., Mossin S. (2020). Examining the effects of nitrogen-doped carbon coating on zinc vanadate nanoflowers towards high performance lithium anode. Electrochim. Acta.

[7] Yin Z., Qin J., Wang W., Cao M. (2017). Rationally designed hollow precursor-derived Zn_3_V_2_O_8_ nanocages as a high-performance anode material for lithium-ion batteries. Nano Energy.

[8] Chandra Sekhar S., Ramulu B., Narsimulu D., Junied Arbaz S., Yu J.S. (2020). Metal–Organic Framework-derived Co_3_V_2_O_8_@CuV_2_O_6_ hybrid architecture as a multifunctional binder-free electrode for Li-Ion Batteries and hybrid Supercapacitors. Small.

[9] Ni S.B., Wang X.H., Zhou G., Yang F., Wang J.M., He D.Y. (2010). Hydrothermal synthesis and magnetic property of Cu_3_(OH)_2_V_2_O_7_·nH_2_O. Mater. Lett..

[10] Yu X., Hu F., Guo Z.-Q., Liu L., Song G.-H., Zhu K. (2022). High-performance Cu_0.95_V_2_O_5_ nanoflowers as cathode materials for aqueous zinc-ion batteries. Rare Met..

[11] Bayat A., Reza Mahjoub A., Amini M. (2018). Facile hydrothermal synthesis of the colloidal hierarchical Volborthite (Cu_3_V_2_O_7_(OH)_2_·2H_2_O) hollow sphere phosphors. J. Lumin..

[12] Fu X., Hu Z.S., Gu G.H., Wang D.B., Zhu H.T., Zhou X.D. (2005). Study on the Preparation of Nano-Materials with Extractant and Extraction Systems. Proceedings of the International Solvent Extraction Conference 2005.

[13] Shi H.Q., Fu X., Zhou X.D., Hu Z.S. (2005). Preparation of Organic Fluids Containing Ag_2_S Nano-Particles with the Extractant Cyanex 301. Proceedings of the International Solvent Extraction Conference 2005.

[14] Zhang S.L., Shi H.Q., Fu X., Hu Z.S. (2005). Preparation and Characterisation of Organic Fluids Containing Bi_2_S_3_ Nano-Particles. Proceedings of the International Solvent Extraction Conference 2005.

[15] Sánchez-Loredo G., Tovar-Tovar R., Aguilera-Mares J., Ruiz F., Martínez-Castañón G., Moyer B. (2008). Stabilized metal and metal sulphide nanoparticles prepared by the two-phase liquid-liquid method. Solvent Extraction: Fundamentals to Industrial Applications.

[16] Konishi Y., Asai S., Murai T., Takemori H. (1997). Preparation of fine ceria powders by hydrolysis of cerium(IV) carboxylate solutions. Metall. Mater. Trans. B.

[17] Bergmann J., Friedel P., Kleeberg R. (1998). BGMN—A new fundamental parameters-based Rietveld program for laboratory X-ray sources, its use in quantitative analysis and structure investigations. CPD Newslett. Commiss. Powder Diffract. Int. Union Crystallogr..

[18] Döbelin N., Kleeberg R. (2015). Profex: A graphical user interface for the Rietveld refinement program BGMN. J. Appl. Crystallogr..

[19] Sánchez-Loredo M.G., Palomares-Sánchez S.A., Labrada-Delgado G.J., Helbig T., Chekhonin P., Ebert D., Möckel R., Owusu Afriyie J., Kelly N. (2022). Preparation of volborthite by a facile synthetic chemical solvent extraction method. Nanomaterials.

[20] Kasatkin A.V., Plášil J., Pekov I.V., Belakovskiy D.I., Nestola F., Čejka J., Vigasina M.F., Zorzi F., Thorne B. (2015). Karpenkoite, Co_3_(V_2_O_7_)(OH)_2_·2H_2_O, a cobalt analogue of martyite from the Little Eva mine, Grand County, Utah, USA. J. Geosci..

[21] Naz G., Othaman Z., Shamsuddin M., Krishna Ghoshal S. (2016). Aliquat 336 stabilized multi-faceted gold nanoparticles with minimal ligand density. Appl. Surf. Sci..

[22] Le M.N., Son S.H., Lee M.S. (2019). Extraction behavior of hydrogen ion by an ionic liquid mixture of Aliquat 336 and Cyanex 272 in chloride solution. Korean J. Met. Mater..

[23] Nguyen V.N.H., Le M.N., Lee M.S. (2020). Comparison of extraction ability between a mixture of Alamine 336/Aliquat 336 and D2EHPA and ionic liquid ALi-D2 from weak hydrochloric acid solution. Metals.

[24] Bülbül B., Beyaz S., Akyol M., Ekicibil A. (2020). Simple manufacturing and metal type-dependent properties of M_3_(OH)_2_V_2_O_7_·nH_2_O (M, Co, Ni, Cu, Zn) nanostructures. Nanochem. Res..

[25] Zhang S.Y., Sun Y., Li C.S., Hua R.S. (2013). Rational synthesis of copper vanadates/polypyrrole nanowires with enhanced electrochemical property. Mater. Lett..

[26] Zhang S.Y., Xiao X., Lu M., Li Z.Q. (2013). Zn_3_V_2_O_7_(OH)_2_.2H_2_O and Zn_3_(VO_4_)_2_ 3D microspheres as anode materials for lithium-ion batteries. J. Mater. Sci..

[27] Liu X., Qin B., Zhang H., Moretti A., Passerini S. (2019). Glyme-Based Electrolyte for Na/Bilayered V_2_O_5_ Batteries. ACS Appl. Energy Mater..

[28] Liu X., Li Q., Ma K., Yang G., Wang C. (2019). Graphene oxide wrapped CuV_2_O_6_ nanobelts as high-capacity and long-life cathode materials of aqueous zinc-ion batteries. ACS Nano.

[29] Liu C., Tian M., Wang M., Zheng J., Wang S., Yan M., Wang Z., Yin Z., Yang J., Cao G. (2020). Catalyzing zinc-ion intercalation in hydrated vanadates for aqueous zinc-ion batteries. J. Mater. Chem. A.

[30] Giorgetti M., Mukerjee S., Passerini S., McBreen J., Smyr W.H. (2001). Evidence for reversible formation of metallic Cu Cu_0.1_V_2_O_5_ xerogel cathodes during intercalation cycling of Li^+ ^ ions as detected by X-ray absorption spectroscopy. J. Electrochem. Soc..

[31] Liu X., Zarrabeitia M., Qin B., Elia G.A., Passerini S. (2020). Cathode-Electrolyte interphase in a LiTFSI/Tetraglyme electrolyte promoting the cyclability of V_2_O_5_. ACS Appl. Mater. Interfaces.

