# Supplementary material for: Precipitation Stripping of V(V) as a Novel Approach for the Preparation of Two-Dimensional Transition Metal Vanadates
Source: Nanomaterials (Basel). 2023 Dec 22;14(1):38. doi: 10.3390/nano14010038 (PMC10780767; doi:10.3390/nano14010038)
Supplement: Supplementary file 1 [file nanomaterials-14-00038-s001.zip › nanomaterials-2765273-supplementary.pdf]

Electronic Supporting Information (ESI)

Precipitation Stripping of V(V) as a Novel Approach for the Preparation of Two-Dimensional Transition Metal Vanadates

María Guadalupe Sánchez-Loredo [1,2,*], Paul Chekhonin [3], Doreen Ebert [1], Ulrike Fischer [1], Xu Liu [4,5], Robert Möckel [1], Gladis Judith Labrada-Delgado [6], Stefano Passerini [4,5,7] and Norman Kelly [1]

[1] Helmholtz-Zentrum Dresden-Rossendorf (HZDR), Helmholtz-Institut Freiberg für Ressourcentechnologie (HIF), Chemnitzer Str. 40, 09599 Freiberg, Germany; d.ebert@hzdr.de (D.E.); u.fischer@hzdr.de (U.F.); r.moeckel@hzdr.de (R.M.); n.kelly@hzdr.de (N.K.)
[2] Instituto de Metalurgia, Facultad de Ingeniería, Universidad Autónoma de San Luis Potosí, Sierra Leona 550, San Luis Potosí 78210, Mexico
[3] Helmholtz-Zentrum Dresden-Rossendorf, Institut für Ressourcenökologie, Bautzner Landstraße 400, 01328 Dresden, Germany; p.chekhonin@hzdr.de
[4] Helmholtz Institute Ulm (HIU), Helmholtzstraße 11, 89081 Ulm, Germany; xu.liu@kit.edu (X.L.); stefano.passerini@kit.edu (S.P.)
[5] Karlsruhe Institute of Technology (KIT), P.O. Box 3640, 76021 Karlsruhe, Germany
[6] Instituto Potosino de Investigación Científica y Tecnológica, Camino a la Presa San José 2055, San Luis Potosí 78216, Mexico; gladis.labrada@ipicyt.edu.mx
[7] Chemistry Department, Sapienza University of Rome, Piazzale Aldo Moro 5, I-00185 Rome, Italy
* Correspondence: msanchez@uaslp.mx

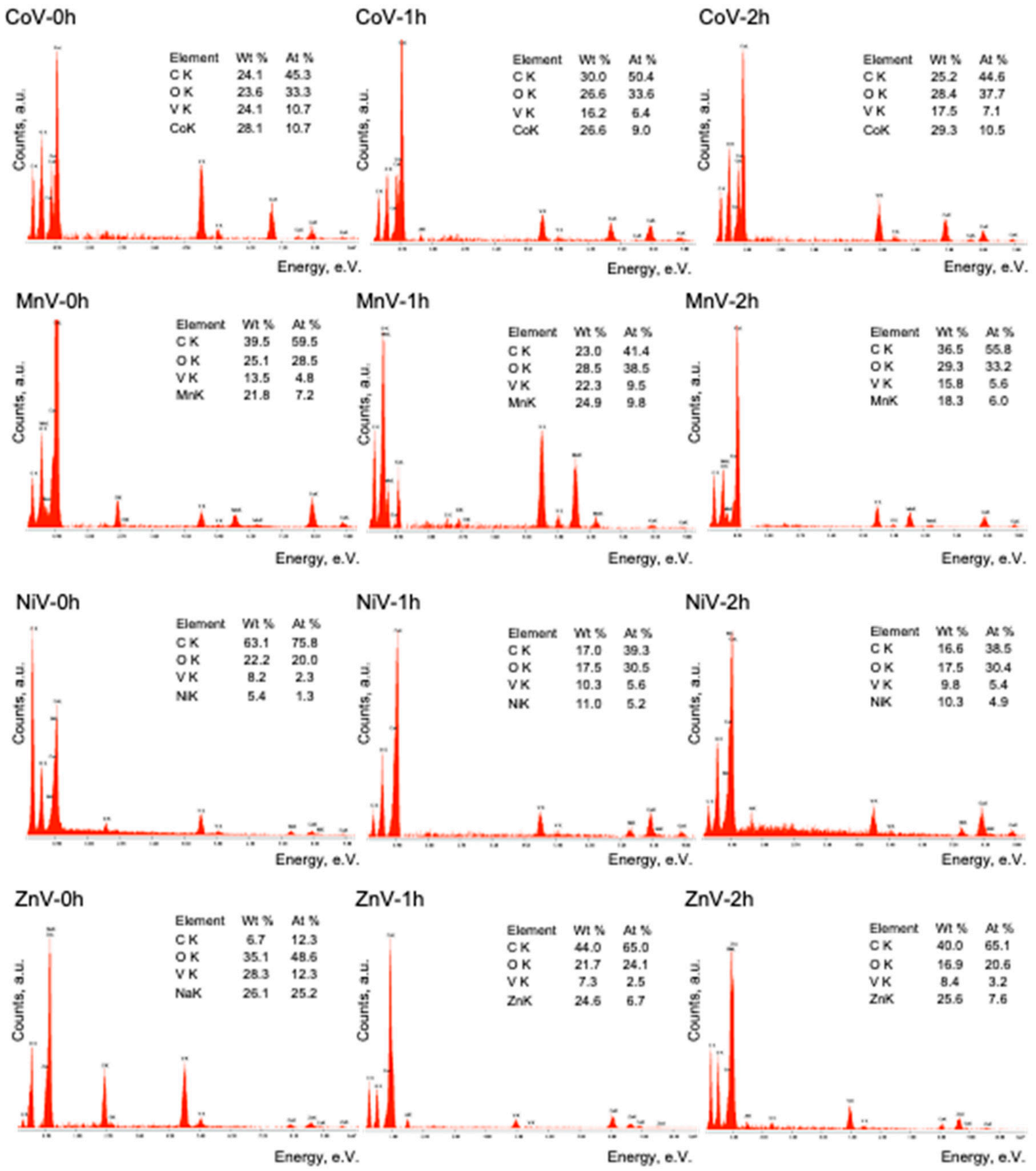

Figure S1. EDS spectra of the metal vanadates obtained at different addition times

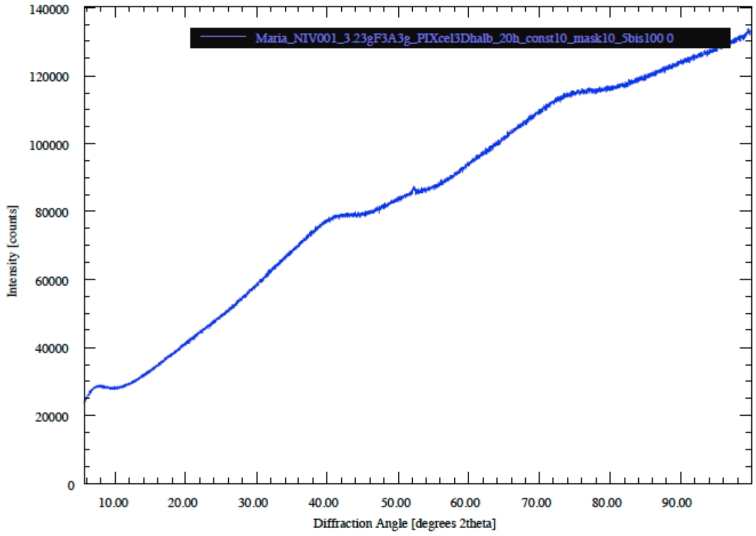

Figure S2. XRD pattern of nickel vanadate product (addition time 1 h)

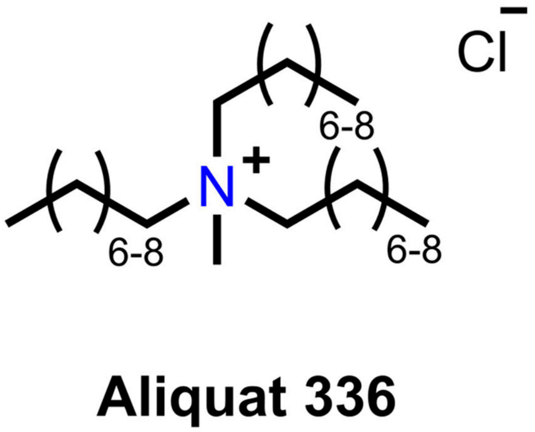

Figure S3. Structure of Aliquat®336

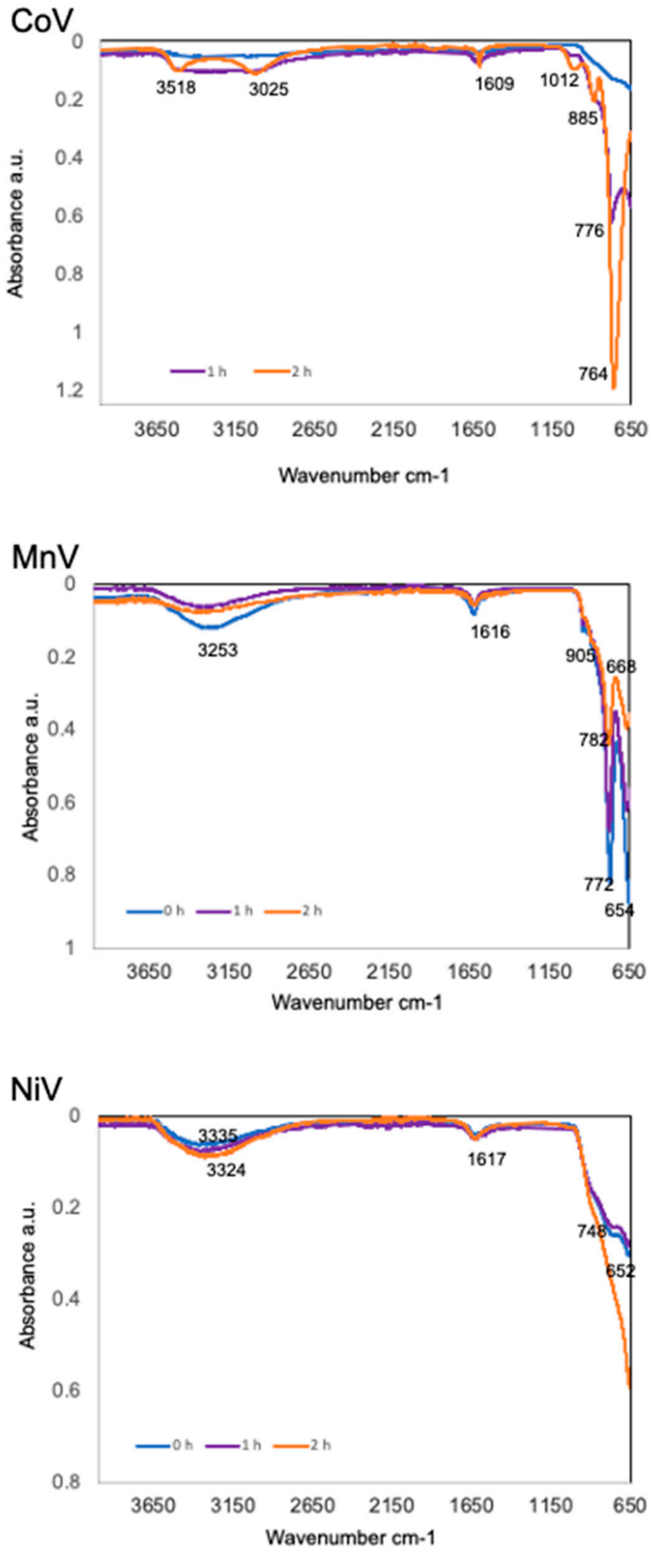

Figure S4. ATR spectra of cobalt, manganese and nickel vanadate powders prepared by precipitation stripping from Aliquat 336 organic solutions
